# P-1755. Impact of A Pharmacist Vancomycin Dosing and Monitoring Consult Service in The Medical Intensive Care Unit

**DOI:** 10.1093/ofid/ofae631.1918

**Published:** 2025-01-29

**Authors:** Krysten McNaught, Jason Hedvat, Young Lee, David C Perlman, Sanjana Koshy, Aniket Sharma, Amanda Devlin

**Affiliations:** UF Health Shands Hospital, gainesville, Florida; Mount Sinai Beth Israel, New York, New York; Mount Sinai Beth Israel, New York, New York; Icahn School of Medicine at Mount Sinai, New York, New York; Mount Sinai Beth Israel, New York, New York; Mount Sinai Hospital, New York, New York; Mount Sinai Beth Israel, New York, New York

## Abstract

**Background:**

Pharmacist-led vancomycin dosing and monitoring is associated with significant decreases in acute kidney injury (AKI), improvement in treatment efficacy, and reduced mortality. Critically ill patients experience variability of vancomycin pharmacokinetics and pharmacodynamics and require increased monitoring. This study aimed to compare patient outcomes before and following the implementation of a pharmacist consult vancomycin dosing and monitoring service in critically ill patients.

**Methods:**

This was a pre- and post-study conducted in a 16-bed medical intensive care unit (MICU) at Mount Sinai Beth Israel. All adult patients who received vancomycin from January 1, 2022, to June 30, 2022, in the pre-group and from February 21, 2023, to May 23, 2023, in the post-group were included. The primary outcome was the proportion of patients within therapeutic trough range at 72 hours. Secondary outcomes included the proportion of patients with AKI, supra-therapeutic levels, sub-therapeutic levels, appropriate initial level time, dose, and frequency. A therapeutic level was defined as 10-15 mg/L for urinary tract and skin and soft tissue infections or 15-20 mg/L for bacteremia, pneumonia, meningitis, endocarditis, and osteomyelitis. Comparisons were performed using Chi-squared test. P < .05 was considered statistically significant.

**Results:**

Ninety-six patients were included in the final analysis; 53 patients in the pre-group and 43 patients in the post-group. The most common indication for vancomycin was pneumonia (64%). No statistically significant difference was found for the primary outcome between the two groups (43% vs. 41%, P = .8). The proportion of patients with supra-therapeutic levels or with AKI at 72 hours was significantly higher in the pre- group vs. the post-group (52% vs. 12%; P = .0007) and (57% vs. 30%; P = .001) respectively. Patients in the pre-group vs. the post-group were significantly less likely to have an appropriate initial level time (57% vs. 79%; P = .03) and initial vancomycin frequency (51% vs. 86%; P = .0003). No significant differences were found in any of the other secondary endpoints.

**Conclusion:**

A pharmacist consult vancomycin dosing and monitoring service significantly reduced the incidence of AKI and supra-therapeutic vancomycin levels in MICU patients.

**Disclosures:**

**All Authors**: No reported disclosures

